# Crystal structure of (*E*)-3-(2,4-di­meth­oxy­phen­yl)-1-(1-hy­droxy­naphthalen-2-yl)prop-2-en-1-one

**DOI:** 10.1107/S1600536814018704

**Published:** 2014-08-23

**Authors:** Dongsoo Koh

**Affiliations:** a Department of Applied Chemistry, Dongduk Women’s University, Seoul 136-714, Republic of Korea

**Keywords:** crystal structure, chalcone, enone, benzochalcone, naphthalene

## Abstract

In the title compound, C_21_H_18_O_4_, the C=C bond of the central enone group adopts an *E* conformation. The dihedral angle formed by the benzene ring and the naphthalene ring system is 6.60 (2)°. The meth­oxy groups on the benzene ring are essentially coplanar with the ring; the C—C—O—C torsion angles being 1.6 (2) and −177.1 (1)°. The hy­droxy group attached to the naphthalene ring is involved in an intra­molecular O—H⋯O hydrogen bond. The relative conformation of the two double bonds in the enone group is *s-cisoid*. In the crystal, weak C—H⋯O hydrogen bonds link the mol­ecules into chains propagating along [010].

## Related literature   

For the synthesis and biological properties of chalcone derivatives, see: Fuchigami *et al.* (2014 ▶); Kim *et al.* (2014 ▶); Mai *et al.* (2014 ▶); Smit & D′Na (2014 ▶). For details concerning benzochalcone derivatives, see: Juvale *et al.* (2013 ▶); Shin *et al.* (2013 ▶). For related structures, see: Ahn *et al.* (2013 ▶); Lim & Koh (2013 ▶).
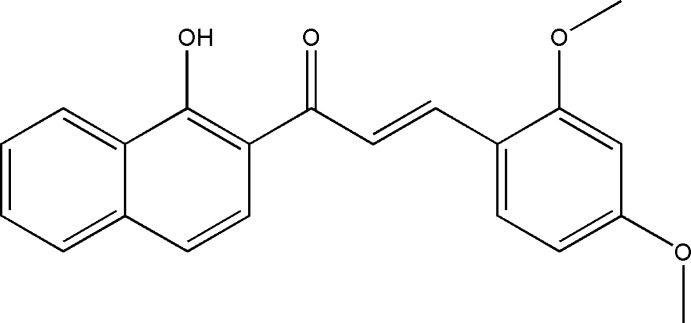


## Experimental   

### Crystal data  

C_21_H_18_O_4_
*M* *_r_* = 334.35
Monoclinic, 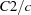

*a* = 14.1148 (9) Å
*b* = 14.7489 (9) Å
*c* = 15.5929 (10) Å
β = 92.804 (4)°
*V* = 3242.2 (4) Å^3^
*Z* = 8
Cu *K*α radiation
μ = 0.77 mm^−1^
*T* = 147 K
0.19 × 0.12 × 0.08 mm

### Data collection  

Bruker Kappa APEX DUO CCD diffractometer
Absorption correction: multi-scan (*SADABS*; Bruker, 2007 ▶) *T*_min_ = 0.669, *T*_max_ = 0.753
8800 measured reflections
2755 independent reflections
2505 reflections with *I* > 2σ(*I*)
*R*_int_ = 0.026

### Refinement  

*R*[*F* ^2^ > 2σ(*F* ^2^)] = 0.036
*wR*(*F* ^2^) = 0.095
*S* = 1.05
2755 reflections
232 parameters
H atoms treated by a mixture of independent and constrained refinement
Δρ_max_ = 0.15 e Å^−3^
Δρ_min_ = −0.27 e Å^−3^

### 

Data collection: *APEX2* (Bruker, 2007 ▶); cell refinement: *SAINT* (Bruker, 2007 ▶); data reduction: *SAINT*; program(s) used to solve structure: *SHELXS97* (Sheldrick, 2008 ▶); program(s) used to refine structure: *SHELXL97* (Sheldrick, 2008 ▶); molecular graphics: *PLATON* (Spek, 2009 ▶); software used to prepare material for publication: *SHELXTL* (Sheldrick, 2008 ▶).

## Supplementary Material

Crystal structure: contains datablock(s) I, New_Global_Publ_Block. DOI: 10.1107/S1600536814018704/su2770sup1.cif

Structure factors: contains datablock(s) I. DOI: 10.1107/S1600536814018704/su2770Isup2.hkl

Supporting information file. DOI: 10.1107/S1600536814018704/su2770Isup3.cml

**Fig. 1.**
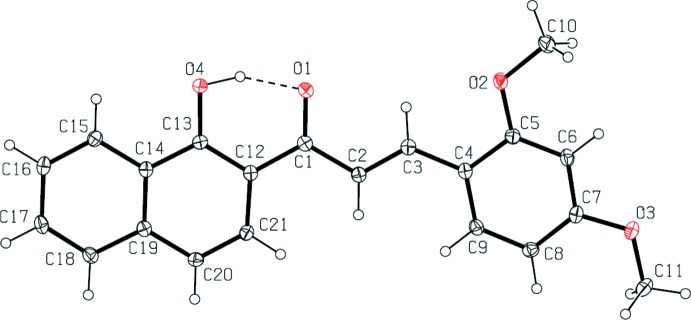
The molecular structure of the title molecule, with atom labelling. The displacement ellipsoids are drawn at the 50% probability level

**Fig. 2.**
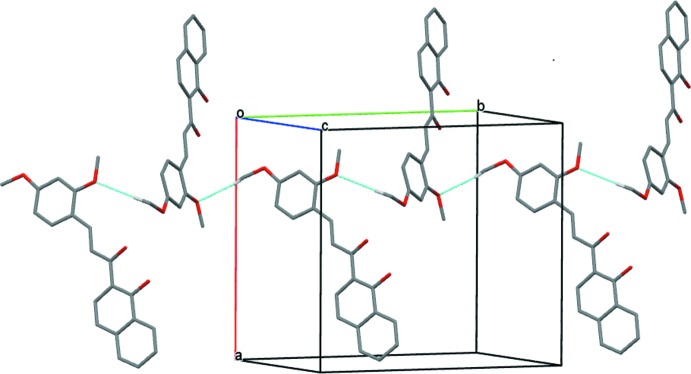
A partial view along the c axis of the crystal packing of the title compound. The C—H···O hydrogen bonds are shown as dashed lines (see Table 1 for details; H atoms not involved in hydrogen bonding have been omitted for clarity).

. DOI: 10.1107/S1600536814018704/su2770fig1.tif
The mol­ecular structure of the title mol­ecule, with atom labelling. The displacement ellipsoids are drawn at the 50% probability level

c . DOI: 10.1107/S1600536814018704/su2770fig2.tif
A partial view along the *c* axis of the crystal packing of the title compound. The C—H⋯O hydrogen bonds are shown as dashed lines (see Table 1 for details; H atoms not involved in hydrogen bonding have been omitted for clarity).

CCDC reference: 1019819

Additional supporting information:  crystallographic information; 3D view; checkCIF report

## Figures and Tables

**Table 1:** Hydrogen-bond geometry (Å, °)

*D*—H⋯*A*	*D*—H	H⋯*A*	*D*⋯*A*	*D*—H⋯*A*
O4—H4*O*⋯O1	0.97 (2)	1.59 (2)	2.499 (1)	155.1 (19)
C10—H10*A*⋯O1^i^	0.98	2.57	3.538 (2)	167
C11—H11*B*⋯O2^ii^	0.98	2.58	3.442 (2)	147

Symmetry codes: (i) 
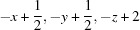
; (ii) 
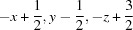
.

## supplementary crystallographic information

### S1. Synthesis and crystallization

To a solution of 2,4-di­meth­oxy­benzaldehyde (830 mg, 5 mmol) in 50 ml of ethanol was added 1-hy­droxy-2-aceto­naphthone (930 mg, 1 mmol) and the temperature was adjusted to around 275–276 K in an ice-bath. To the cooled reaction mixture 5 ml of 50% aqueous KOH solution was added, and the reaction mixture was stirred at room temperature for 24 h. This mixture was poured into iced water (100 ml) and was acidified (pH = 3) with 6 N HCl solution to give a precipitate. Filtration and washing with water afforded the crude solid of the title compound (520 mg, 31%). Recrystallization of the solid from ethanol gave single crystals crystals which were suitable for X-ray diffraction (M.p. 434–435 K).

### S2. Refinement details

The OH H atom was located in a difference Fourier map and freely refined. The C-bound H atoms were placed in calculated positions and refined as riding atoms: C–H = 0.95 - 0.98 Å with *U*_iso_(H) = 1.5*U*_eq_(C-methyl) and = 1.2U_eq_(C) for other H atoms.

### Crystal data

**Table:**

C_21_H_18_O_4_	*F*(000) = 1408
*M**_r_* = 334.35	*D*_x_ = 1.370 Mg m^−^^3^
Monoclinic, *C*2/*c*	Cu *K*α radiation, λ = 1.54178 Å
Hall symbol: -C 2yc	Cell parameters from 4341 reflections
*a* = 14.1148 (9) Å	θ = 4.3–66.4°
*b* = 14.7489 (9) Å	µ = 0.77 mm^−^^1^
*c* = 15.5929 (10) Å	*T* = 147 K
β = 92.804 (4)°	Needle, orange
*V* = 3242.2 (4) Å^3^	0.19 × 0.12 × 0.08 mm
*Z* = 8	

### Data collection

**Table:**

Bruker Kappa APEX DUO CCD diffractometer	2755 independent reflections
Radiation source: Bruker ImuS	2505 reflections with *I* > 2σ(*I*)
Multi-layer optics monochromator	*R*_int_ = 0.026
φ and ω scans	θ_max_ = 66.5°, θ_min_ = 4.3°
Absorption correction: multi-scan (*SADABS*; Bruker, 2007)	*h* = −16→16
*T*_min_ = 0.669, *T*_max_ = 0.753	*k* = −17→17
8800 measured reflections	*l* = −13→18

### Refinement

**Table:**

Refinement on *F*^2^	Primary atom site location: structure-invariant direct methods
Least-squares matrix: full	Secondary atom site location: difference Fourier map
*R*[*F*^2^ > 2σ(*F*^2^)] = 0.036	Hydrogen site location: inferred from neighbouring sites
*wR*(*F*^2^) = 0.095	H atoms treated by a mixture of independent and constrained refinement
*S* = 1.05	*w* = 1/[σ^2^(*F*_o_^2^) + (0.049*P*)^2^ + 1.958*P*] where *P* = (*F*_o_^2^ + 2*F*_c_^2^)/3
2755 reflections	(Δ/σ)_max_ = 0.001
232 parameters	Δρ_max_ = 0.15 e Å^−^^3^
0 restraints	Δρ_min_ = −0.27 e Å^−^^3^

### Special details

**Table:**

Geometry. All e.s.d.'s (except the e.s.d. in the dihedral angle between two l.s. planes) are estimated using the full covariance matrix. The cell e.s.d.'s are taken into account individually in the estimation of e.s.d.'s in distances, angles and torsion angles; correlations between e.s.d.'s in cell parameters are only used when they are defined by crystal symmetry. An approximate (isotropic) treatment of cell e.s.d.'s is used for estimating e.s.d.'s involving l.s. planes.
Refinement. Refinement of *F*^2^ against ALL reflections. The weighted *R*-factor *wR* and goodness of fit *S* are based on *F*^2^, conventional *R*-factors *R* are based on *F*, with *F* set to zero for negative *F*^2^. The threshold expression of *F*^2^ > σ(*F*^2^) is used only for calculating *R*-factors(gt) *etc*. and is not relevant to the choice of reflections for refinement. *R*-factors based on *F*^2^ are statistically about twice as large as those based on *F*, and *R*- factors based on ALL data will be even larger.

### Fractional atomic coordinates and isotropic or equivalent isotropic displacement parameters (Å^2^)

**Table:**

	*x*	*y*	*z*	*U*_iso_*/*U*_eq_	
O1	0.46036 (6)	0.17828 (6)	1.03233 (6)	0.0275 (2)	
O2	0.19523 (6)	0.11183 (7)	0.85783 (7)	0.0345 (3)	
O3	0.17234 (6)	−0.10692 (6)	0.63594 (6)	0.0266 (2)	
O4	0.59612 (6)	0.24290 (6)	1.12130 (6)	0.0251 (2)	
C1	0.51556 (9)	0.13224 (8)	0.98872 (8)	0.0210 (3)	
C2	0.47622 (9)	0.07880 (8)	0.91593 (8)	0.0223 (3)	
H2A	0.5173	0.0432	0.8832	0.027*	
C3	0.38286 (9)	0.07983 (8)	0.89525 (8)	0.0213 (3)	
H3A	0.3454	0.1177	0.9294	0.026*	
C4	0.33198 (8)	0.03012 (8)	0.82693 (8)	0.0201 (3)	
C5	0.23466 (9)	0.04712 (8)	0.80855 (8)	0.0226 (3)	
C6	0.18419 (9)	0.00040 (9)	0.74439 (8)	0.0240 (3)	
H6A	0.1190	0.0134	0.7324	0.029*	
C7	0.22926 (9)	−0.06572 (9)	0.69744 (8)	0.0215 (3)	
C8	0.32474 (9)	−0.08523 (9)	0.71418 (8)	0.0233 (3)	
H8A	0.3557	−0.1302	0.6822	0.028*	
C9	0.37358 (9)	−0.03735 (9)	0.77878 (8)	0.0222 (3)	
H9A	0.4386	−0.0512	0.7908	0.027*	
C10	0.09670 (11)	0.13031 (13)	0.84355 (13)	0.0516 (5)	
H10A	0.0772	0.1763	0.8845	0.077*	
H10B	0.0603	0.0746	0.8514	0.077*	
H10C	0.0847	0.1528	0.7849	0.077*	
C11	0.21279 (10)	−0.17857 (10)	0.58794 (9)	0.0339 (3)	
H11A	0.1648	−0.2029	0.5466	0.051*	
H11B	0.2349	−0.2269	0.6272	0.051*	
H11C	0.2665	−0.1551	0.5571	0.051*	
C12	0.61749 (8)	0.13277 (8)	1.01100 (8)	0.0193 (3)	
C13	0.65294 (8)	0.18928 (8)	1.07709 (7)	0.0190 (3)	
C14	0.75210 (8)	0.19224 (8)	1.10073 (7)	0.0193 (3)	
C15	0.78867 (9)	0.25063 (8)	1.16617 (8)	0.0220 (3)	
H15A	0.7470	0.2893	1.1952	0.026*	
C16	0.88385 (9)	0.25175 (9)	1.18786 (8)	0.0251 (3)	
H16A	0.9081	0.2916	1.2315	0.030*	
C17	0.94584 (9)	0.19415 (9)	1.14567 (8)	0.0259 (3)	
H17A	1.0117	0.1946	1.1616	0.031*	
C18	0.91205 (9)	0.13741 (9)	1.08185 (8)	0.0246 (3)	
H18A	0.9549	0.0991	1.0537	0.030*	
C19	0.81422 (9)	0.13491 (8)	1.05720 (8)	0.0203 (3)	
C20	0.77706 (9)	0.07710 (9)	0.99071 (8)	0.0228 (3)	
H20A	0.8185	0.0382	0.9617	0.027*	
C21	0.68288 (9)	0.07699 (8)	0.96827 (8)	0.0216 (3)	
H21A	0.6599	0.0387	0.9229	0.026*	
H4O	0.5337 (15)	0.2284 (14)	1.0965 (13)	0.059 (6)*	

### Atomic displacement parameters (Å^2^)

**Table:**

	*U*^11^	*U*^22^	*U*^33^	*U*^12^	*U*^13^	*U*^23^
O1	0.0193 (4)	0.0320 (5)	0.0312 (5)	0.0025 (4)	−0.0004 (4)	−0.0075 (4)
O2	0.0217 (5)	0.0350 (6)	0.0455 (6)	0.0096 (4)	−0.0106 (4)	−0.0179 (5)
O3	0.0286 (5)	0.0265 (5)	0.0242 (5)	−0.0033 (4)	−0.0046 (4)	−0.0038 (4)
O4	0.0202 (5)	0.0282 (5)	0.0267 (5)	0.0035 (4)	−0.0002 (4)	−0.0078 (4)
C1	0.0217 (6)	0.0192 (6)	0.0221 (6)	−0.0003 (5)	0.0010 (5)	0.0028 (5)
C2	0.0208 (6)	0.0227 (6)	0.0234 (6)	−0.0006 (5)	0.0006 (5)	−0.0010 (5)
C3	0.0229 (6)	0.0184 (6)	0.0224 (6)	−0.0006 (5)	−0.0001 (5)	0.0019 (5)
C4	0.0206 (6)	0.0188 (6)	0.0208 (6)	−0.0020 (5)	−0.0004 (5)	0.0037 (5)
C5	0.0228 (6)	0.0193 (6)	0.0254 (6)	0.0018 (5)	−0.0015 (5)	0.0003 (5)
C6	0.0207 (6)	0.0238 (7)	0.0270 (7)	0.0004 (5)	−0.0052 (5)	0.0009 (5)
C7	0.0251 (6)	0.0212 (6)	0.0179 (6)	−0.0055 (5)	−0.0019 (5)	0.0038 (5)
C8	0.0251 (6)	0.0234 (7)	0.0217 (6)	−0.0009 (5)	0.0046 (5)	0.0000 (5)
C9	0.0187 (6)	0.0255 (7)	0.0224 (6)	−0.0019 (5)	0.0012 (5)	0.0026 (5)
C10	0.0254 (8)	0.0575 (11)	0.0703 (12)	0.0182 (7)	−0.0136 (7)	−0.0335 (9)
C11	0.0387 (8)	0.0336 (8)	0.0291 (7)	−0.0041 (6)	−0.0006 (6)	−0.0114 (6)
C12	0.0201 (6)	0.0181 (6)	0.0197 (6)	−0.0007 (5)	−0.0004 (5)	0.0023 (5)
C13	0.0218 (6)	0.0168 (6)	0.0184 (6)	0.0011 (5)	0.0020 (5)	0.0020 (5)
C14	0.0209 (6)	0.0189 (6)	0.0178 (6)	−0.0012 (5)	−0.0009 (5)	0.0037 (5)
C15	0.0249 (6)	0.0215 (6)	0.0195 (6)	−0.0004 (5)	−0.0003 (5)	0.0008 (5)
C16	0.0266 (7)	0.0264 (7)	0.0218 (6)	−0.0045 (5)	−0.0051 (5)	0.0008 (5)
C17	0.0196 (6)	0.0320 (7)	0.0254 (6)	−0.0019 (5)	−0.0043 (5)	0.0039 (5)
C18	0.0204 (6)	0.0277 (7)	0.0255 (7)	0.0022 (5)	0.0000 (5)	0.0020 (5)
C19	0.0211 (6)	0.0207 (6)	0.0191 (6)	0.0001 (5)	−0.0004 (5)	0.0045 (5)
C20	0.0223 (6)	0.0224 (6)	0.0237 (6)	0.0030 (5)	0.0019 (5)	−0.0023 (5)
C21	0.0231 (6)	0.0204 (6)	0.0211 (6)	−0.0008 (5)	−0.0012 (5)	−0.0017 (5)

### Geometric parameters (Å, º)

**Table:**

O1—C1	1.2580 (15)	C10—H10B	0.9800
O2—C5	1.3612 (16)	C10—H10C	0.9800
O2—C10	1.4239 (17)	C11—H11A	0.9800
O3—C7	1.3635 (15)	C11—H11B	0.9800
O3—C11	1.4299 (17)	C11—H11C	0.9800
O4—C13	1.3408 (15)	C12—C13	1.3987 (17)
O4—H4O	0.97 (2)	C12—C21	1.4258 (17)
C1—C12	1.4634 (17)	C13—C14	1.4306 (17)
C1—C2	1.4683 (18)	C14—C15	1.4133 (17)
C2—C3	1.3414 (18)	C14—C19	1.4152 (17)
C2—H2A	0.9500	C15—C16	1.3693 (18)
C3—C4	1.4537 (17)	C15—H15A	0.9500
C3—H3A	0.9500	C16—C17	1.4059 (19)
C4—C9	1.3936 (18)	C16—H16A	0.9500
C4—C5	1.4119 (17)	C17—C18	1.3680 (19)
C5—C6	1.3832 (18)	C17—H17A	0.9500
C6—C7	1.3919 (18)	C18—C19	1.4156 (17)
C6—H6A	0.9500	C18—H18A	0.9500
C7—C8	1.3902 (18)	C19—C20	1.4227 (18)
C8—C9	1.3858 (18)	C20—C21	1.3580 (17)
C8—H8A	0.9500	C20—H20A	0.9500
C9—H9A	0.9500	C21—H21A	0.9500
C10—H10A	0.9800		
			
C5—O2—C10	117.97 (11)	O3—C11—H11A	109.5
C7—O3—C11	117.44 (10)	O3—C11—H11B	109.5
C13—O4—H4O	102.7 (12)	H11A—C11—H11B	109.5
O1—C1—C12	119.77 (11)	O3—C11—H11C	109.5
O1—C1—C2	119.13 (11)	H11A—C11—H11C	109.5
C12—C1—C2	121.11 (11)	H11B—C11—H11C	109.5
C3—C2—C1	120.65 (11)	C13—C12—C21	118.15 (11)
C3—C2—H2A	119.7	C13—C12—C1	119.35 (11)
C1—C2—H2A	119.7	C21—C12—C1	122.50 (11)
C2—C3—C4	127.94 (12)	O4—C13—C12	121.93 (11)
C2—C3—H3A	116.0	O4—C13—C14	116.85 (11)
C4—C3—H3A	116.0	C12—C13—C14	121.22 (11)
C9—C4—C5	116.67 (11)	C15—C14—C19	119.82 (11)
C9—C4—C3	123.29 (11)	C15—C14—C13	121.53 (11)
C5—C4—C3	120.00 (11)	C19—C14—C13	118.65 (11)
O2—C5—C6	123.18 (11)	C16—C15—C14	120.33 (12)
O2—C5—C4	115.50 (11)	C16—C15—H15A	119.8
C6—C5—C4	121.32 (12)	C14—C15—H15A	119.8
C5—C6—C7	119.80 (11)	C15—C16—C17	120.18 (12)
C5—C6—H6A	120.1	C15—C16—H16A	119.9
C7—C6—H6A	120.1	C17—C16—H16A	119.9
O3—C7—C8	124.91 (11)	C18—C17—C16	120.48 (12)
O3—C7—C6	114.42 (11)	C18—C17—H17A	119.8
C8—C7—C6	120.67 (11)	C16—C17—H17A	119.8
C9—C8—C7	118.31 (12)	C17—C18—C19	120.94 (12)
C9—C8—H8A	120.8	C17—C18—H18A	119.5
C7—C8—H8A	120.8	C19—C18—H18A	119.5
C8—C9—C4	123.21 (12)	C14—C19—C18	118.24 (11)
C8—C9—H9A	118.4	C14—C19—C20	119.51 (11)
C4—C9—H9A	118.4	C18—C19—C20	122.24 (11)
O2—C10—H10A	109.5	C21—C20—C19	120.66 (11)
O2—C10—H10B	109.5	C21—C20—H20A	119.7
H10A—C10—H10B	109.5	C19—C20—H20A	119.7
O2—C10—H10C	109.5	C20—C21—C12	121.79 (11)
H10A—C10—H10C	109.5	C20—C21—H21A	119.1
H10B—C10—H10C	109.5	C12—C21—H21A	119.1
			
O1—C1—C2—C3	0.91 (18)	C2—C1—C12—C21	−4.86 (18)
C12—C1—C2—C3	−179.00 (11)	C21—C12—C13—O4	−179.06 (10)
C1—C2—C3—C4	−178.69 (11)	C1—C12—C13—O4	0.41 (17)
C2—C3—C4—C9	9.1 (2)	C21—C12—C13—C14	0.81 (17)
C2—C3—C4—C5	−173.07 (12)	C1—C12—C13—C14	−179.73 (10)
C10—O2—C5—C6	1.6 (2)	O4—C13—C14—C15	−1.54 (17)
C10—O2—C5—C4	−178.52 (14)	C12—C13—C14—C15	178.59 (11)
C9—C4—C5—O2	178.52 (11)	O4—C13—C14—C19	178.34 (10)
C3—C4—C5—O2	0.59 (17)	C12—C13—C14—C19	−1.53 (17)
C9—C4—C5—C6	−1.56 (18)	C19—C14—C15—C16	−0.24 (18)
C3—C4—C5—C6	−179.48 (11)	C13—C14—C15—C16	179.64 (11)
O2—C5—C6—C7	−179.17 (12)	C14—C15—C16—C17	−0.57 (19)
C4—C5—C6—C7	0.91 (19)	C15—C16—C17—C18	0.90 (19)
C11—O3—C7—C8	3.18 (17)	C16—C17—C18—C19	−0.38 (19)
C11—O3—C7—C6	−177.12 (11)	C15—C14—C19—C18	0.74 (17)
C5—C6—C7—O3	−179.88 (11)	C13—C14—C19—C18	−179.15 (11)
C5—C6—C7—C8	−0.17 (19)	C15—C14—C19—C20	−179.25 (11)
O3—C7—C8—C9	179.82 (11)	C13—C14—C19—C20	0.86 (17)
C6—C7—C8—C9	0.13 (18)	C17—C18—C19—C14	−0.43 (18)
C7—C8—C9—C4	−0.87 (19)	C17—C18—C19—C20	179.56 (12)
C5—C4—C9—C8	1.56 (18)	C14—C19—C20—C21	0.52 (18)
C3—C4—C9—C8	179.41 (11)	C18—C19—C20—C21	−179.47 (12)
O1—C1—C12—C13	−4.21 (17)	C19—C20—C21—C12	−1.29 (19)
C2—C1—C12—C13	175.70 (11)	C13—C12—C21—C20	0.62 (18)
O1—C1—C12—C21	175.23 (11)	C1—C12—C21—C20	−178.83 (12)

### Hydrogen-bond geometry (Å, º)

**Table:**

*D*—H···*A*	*D*—H	H···*A*	*D*···*A*	*D*—H···*A*
O4—H4*O*···O1	0.97 (2)	1.59 (2)	2.499 (1)	155.1 (19)
C10—H10*A*···O1^i^	0.98	2.57	3.538 (2)	167
C11—H11*B*···O2^ii^	0.98	2.58	3.442 (2)	147

Symmetry codes: (i) −*x*+1/2, −*y*+1/2, −*z*+2; (ii) −*x*+1/2, *y*−1/2, −*z*+3/2.
