# Supplementary figures and images for: Crystal structure of (E)-3-(2,4-di­meth­oxy­phen­yl)-1-(1-hy­droxy­naphthalen-2-yl)prop-2-en-1-one
Source: Acta Crystallogr Sect E Struct Rep Online. 2014 Aug 23;70(Pt 9):o1034–5. doi: 10.1107/S1600536814018704 (PMC4186154; doi:10.1107/S1600536814018704)

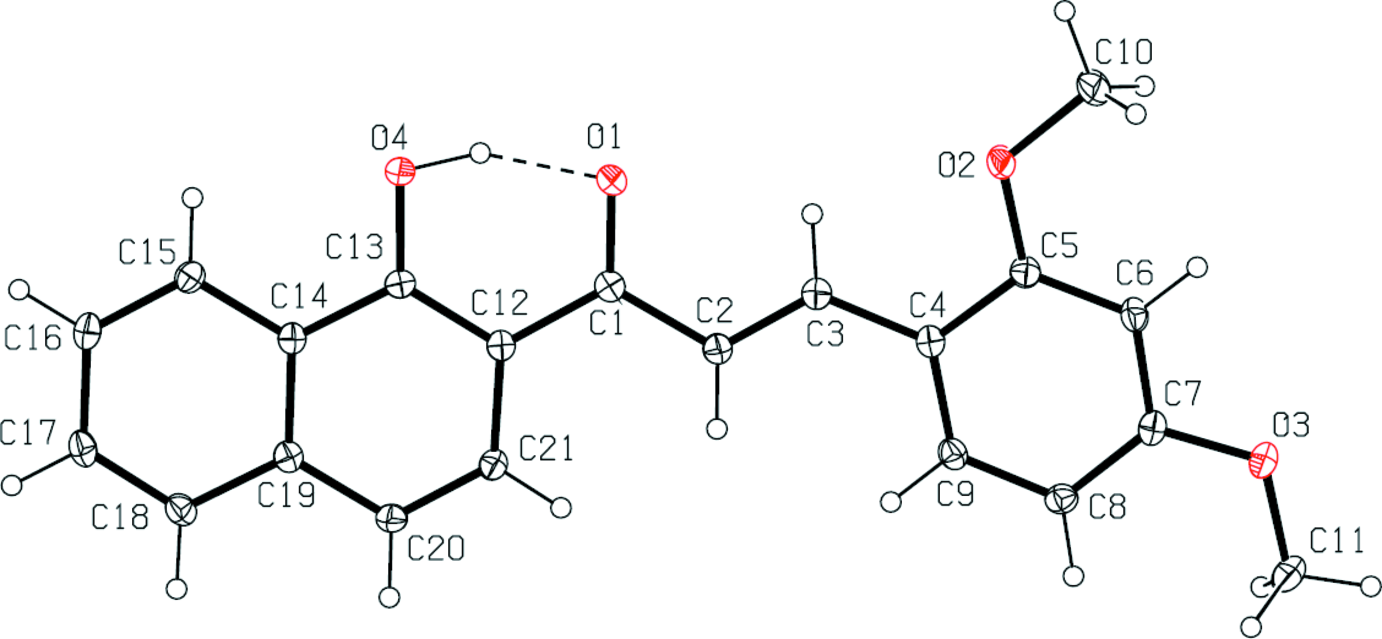

Supplement: Supplementary file 4 [file e-70-o1034-fig1.tif]

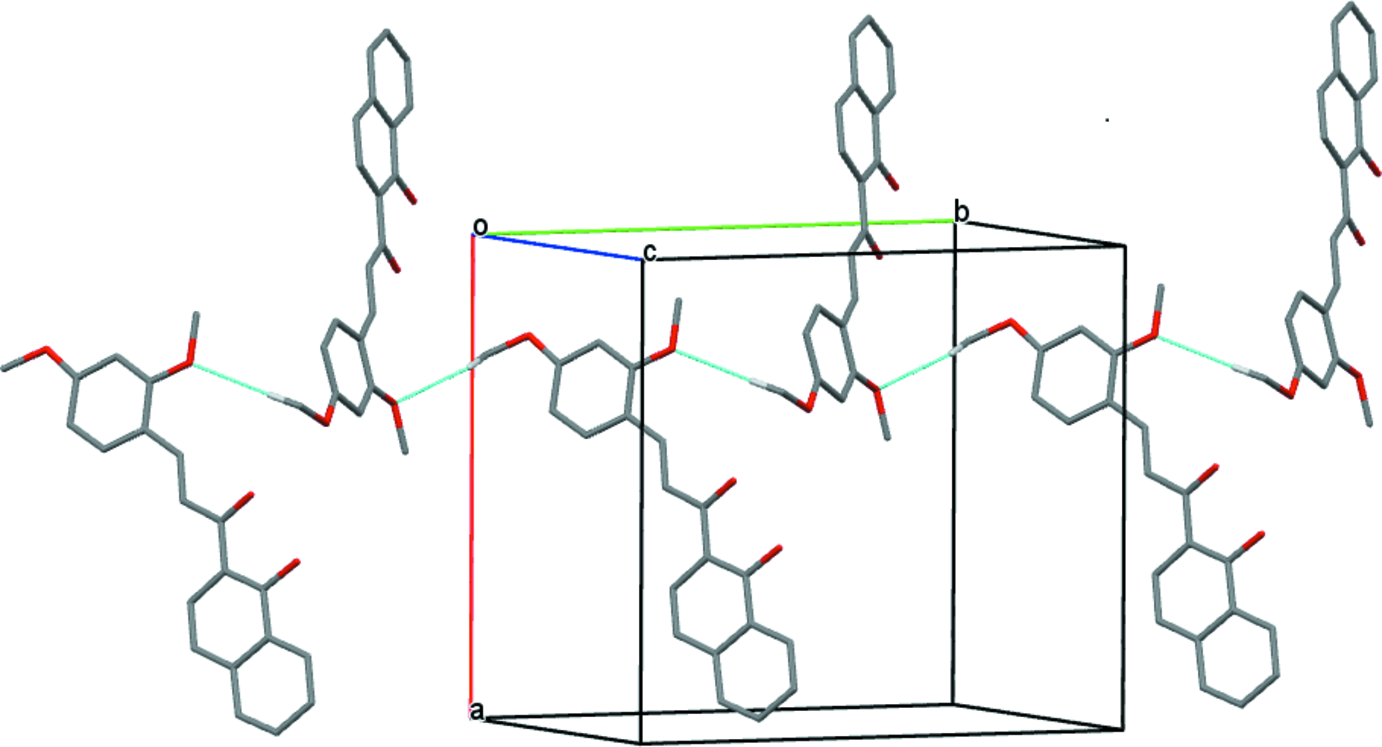

Supplement: Supplementary file 5 [file e-70-o1034-fig2.tif]
